# An overview of the treatments for hypertrophic cardiomyopathy

**DOI:** 10.3389/fcvm.2024.1387596

**Published:** 2024-06-03

**Authors:** Wenna Xu, Fuyu Zhu, Yue Zhang, Peng Li, Yanhui Sheng

**Affiliations:** ^1^ Department of Cardiology, The Affiliated Suzhou Hospital of Nanjing Medical University, Suzhou Municipal Hospital, Gusu School, Nanjing Medical University, Suzhou, Jiangsu, China; ^2^ Department of Cardiology, The First Affiliated Hospital of Nanjing Medical University, Nanjing, Jiangsu, China; ^3^ Key Laboratory of Targeted Intervention of Cardiovascular Disease, Collaborative Innovation Center for Cardiovascular Disease Translational Medicine, Nanjing Medical University, Nanjing, Jiangsu, China; ^4^ Department of Cardiology, Jiangsu Province Hospital, Nanjing, Jiangsu, China

**Keywords:** hypertrophic cardiomyopathy, pathogenesis, pharmacotherapy, mavacamten, invasive treatments, gene therapy

## Abstract

Hypertrophic cardiomyopathy (HCM) is a very prevalent inherited disease with a wide global distribution and a prevalence rate of approximately 0.2% in the general population. Left ventricular hypertrophy (LVH) caused by sarcomere mutation is the primary reason of HCM. The histopathology feature is that cardiomyocyte hypertrophy, myocyte disorder and myocardial fibrosis lead to diminished diastolic function, left ventricular outflow tract obstruction (LVOTO) and arrhythmia, all of which result in serious cardiac complications. Previously, HCM was considered a malignant disease that was almost untreatable. With the improvement of medical standards and increasing awareness of HCM, it has become a highly treatable disease in contemporary times, with a significant decrease in mortality rates. However, there are still significant unmet requirements in the therapy of HCM. This paper draws on more than 100 references from the past four decades and summarizes current advances in the treatment of HCM. The article will review the pathogenesis and types, recent development in pharmacotherapy, invasive treatments and gene therapies, as well as dilemma and future development of HCM.

## 1 Introduction

With an estimated frequency of 0.2% in the general population, HCM is one of the most common inherited cardiovascular disorders (1–3). HCM is typically passed down through autosomal dominant inheritance and is brought on by variations in the sarcomere protein genes (4–6). However, its clinical heterogeneousness and different phenotypic manifestation increase the potentiality of non-genetic or surroundings variables may alter the phenotype of HCM (7). HCM is defined as a maximum left ventricular (LV) wall thickness of more than 15 mm, when there are no abnormal load conditions and no other minor factors (2, 8, 9). Patients typically feel decreased exercise tolerance, heart palpitations, exertional chest discomfort, and dyspnea, mainly due to LVOTO, LV diastolic insufficiency and arrhythmias (7, 10–12). Additionally, the development of atrial fibrillation (AF), heart failure, and sudden cardiac death (SCD) are also significantly influenced by them (7, 13). HCM is, in fact, the most prevalent reason for SCD in young individuals and exercise patients (3, 14).

HCM was formerly thought to be an uncommon, deadly condition with few curative possibilities (15, 16). Effective approaches to care for substantial HCM complications have come about over the last 20 years as medical standards have improved and awareness of HCM has grown. These approaches improve clinical outcomes and significantly reduce mortality and morbidity rates, increasing the likelihood that both adults and children will live healthy, fulfilling lives into their 70s and 90s (15, 16). The overall prognosis for patients with HCM is favorable with available treatments. However, the disease's health burden is also very high (16). This paper summarizes and analyzes the existing treatment methods for HCM, briefly describes the advantages and disadvantages of various existing treatment methods of obstructive HCM (Table 1). And it elaborates recent development in pharmacotherapy, invasive treatments and gene therapies, which provides certain guidance and inspiration for the future research direction and development.

**Table 1:** The advantages and disadvantages of the treatments of obstructive HCM.

Treatment methods		Advantages	Disadvantages
Traditional drugs	β-blockers	Reduce LVOTO (3, 7); improve patient symptoms (3, 7)	May cause bradycardia, hypotension and other adverse reactions (17, 18)
	Verapamil		
	Disopyramide	Delay the need for invasive treatments (19, 20)	Adverse effects such as dry mouth and constipation (21, 22); may cause significant QT interval prolongation (19)
Myosin inhibitors	Mavacamten	Reduce LVOTO (23); increase exercise capacity (23); improve patient symptoms (23)	Significantly lower EF (24–26)
	Aficamten	Shorter half-life (27); wider therapeutic window (27)	Ongoing clinical trials
Invasive treatments	Septal myectomy	Eliminate LVOTO permanently (28); reverse left atrial remodeling (1)	More traumatic; long hospital stays
	PTSMA	Short hospital stays (29, 30); quick recovery (29)	Limited by the structure of the septal coronary (31)
	PIMSRA	Protect the subendocardial conduction system (32)	Lack of long-term follow-up data

LVOTO, left ventricular outflow tract obstruction; EF, ejection fraction; PTSMA, percutaneous transluminal septal myocardial ablation; PIMSRA, percutaneous intramyocardial septal radiofrequency ablation.

## 2 Pathogenesis and types of HCM

In most situations, HCM is inherited as an autosomal dominant characteristic, and about 60% of HCM patients can detect clear pathogenic genes (2, 3, 33). At present, some studies suggest that it is caused by gene mutations encoding structural proteins related to myocardial sarcomere, such as the troponin I, T, the myosin-binding protein C, and the β-myosin heavy chain (2, 3, 10). These gene mutations may cause abnormal structural function of the sarcomere, which may lead to abnormal cardiac contraction, impaired diastolic function and increased energy expenditure, resulting in cardiomyocyte hypertrophy, disarray, myocardial fibrosis, ventricular remodeling, and so on (7). HCM lesions are mainly cardiac hypertrophy, mostly asymmetric hypertrophy, often involving the interventricular septum (IVS) and free wall (3, 11, 34). Ventricular hypertrophy, myocardial fibrosis lead to reduced ventricular compliance, resulting in longer ventricular relaxation time and inadequate ventricular filling, thus leading to ventricular diastolic disorders, secondary regurgitation of the mitral valve, and ischemia of the myocardium (2, 3, 7, 10). The hypertrophic IVS bulges on to the LV, which leads to the narrowing of the left ventricular outflow tract (LVOT), increased blood flow velocity, and forward movement of the anterior mitral leaflet and chordae tendineae, which is called systolic anterior leaflet (SAM), resulting in dynamic obstruction of the LVOT (2, 3, 7). The primary mitral valve related structural abnormalities, such as mitral leaflet elongation and papillary muscle hypertrophy and displacement, also aggravate the LVOTO (2, 3, 7). Obstruction of the LVOT is dynamic, and the degree of obstruction also depends on LV contractility and LV preload conditions (35). Thus, increasing LV contractility (e.g., digitalis drugs) or decreasing return blood flow (e.g., from a squatting to standing position) can increase the degree of LVOTO (35). AF is the most commonly comorbid arrhythmia in HCM (29, 36). LVOTO leads to increased left atrial (LA) afterload, and the LA loses its ability to contract strongly and dilates to remodel triggering mitral valve closure insufficiency, which may lead to electrical remodeling that triggers AF (37–39). LA remodeling also promotes cardiomyocyte fibrosis, which interferes with local atrial conduction, leading to disturbances in electrical activity, which in turn leads to the formation of local conduction refractures as well as conduction blocks, making it more susceptible to the development of AF (40).

According to the characteristics of hemodynamics, patients with HCM can be divided into two different types: obstructive and non-obstructive, depending on the severity of LVOTO (3, 10, 41). (1) Obstructive HCM: this type of HCM has dynamic LVOTO with a LVOT maximum pressure gradient greater than 30 mmHg (3, 34). Resting LVOTO is present in about one-third of HCM patients and is primarily caused by SAM (3, 41). Another third of HCM patients only experience obstruction when provoked (also known as latent obstruction). Patients are without obstruction at rest and with obstruction (maximum pressure gradient greater than 30 mmHg) on stimulation (Valsalva maneuver, inhalation of powerful vasodilators, and exercise treadmill test) (41). (2) Non-obstructive HCM: the remainder of one-third of HCM patients have a maximum pressure LVOT gradient of less than 30 mmHg and no notable obstruction, either at rest or in response to provocation (3, 41). According to the site of myocardial hypertrophy, patients with HCM can be classified into the following types. (1) IVS hypertrophy: it is the most common in clinic, mainly involving the basal of IVS (42). Some of patients can involve the middle IVS, showing IVS hypertrophy at the papillary muscle level in the middle of the LV (29). (2) Apical hypertrophy: it mainly involves the apical part below the level of the papillary muscle of the LV, usually without elevated LVOT gradient (29, 42). (3) Diffuse hypertrophy of LV wall: a few patients show diffuse thickening of the LV wall (29). (4) Biventricular wall hypertrophy: in addition to LV wall hypertrophy, there is also right ventricular (RV) wall hypertrophy (RV free wall thickness >5 mm) (43, 44). (5) Isolated papillary muscle hypertrophy: the main feature is papillary muscle hypertrophy, and the rest of the LV segments are not affected (45).

## 3 Treatment of HCM

**Figure 1:**
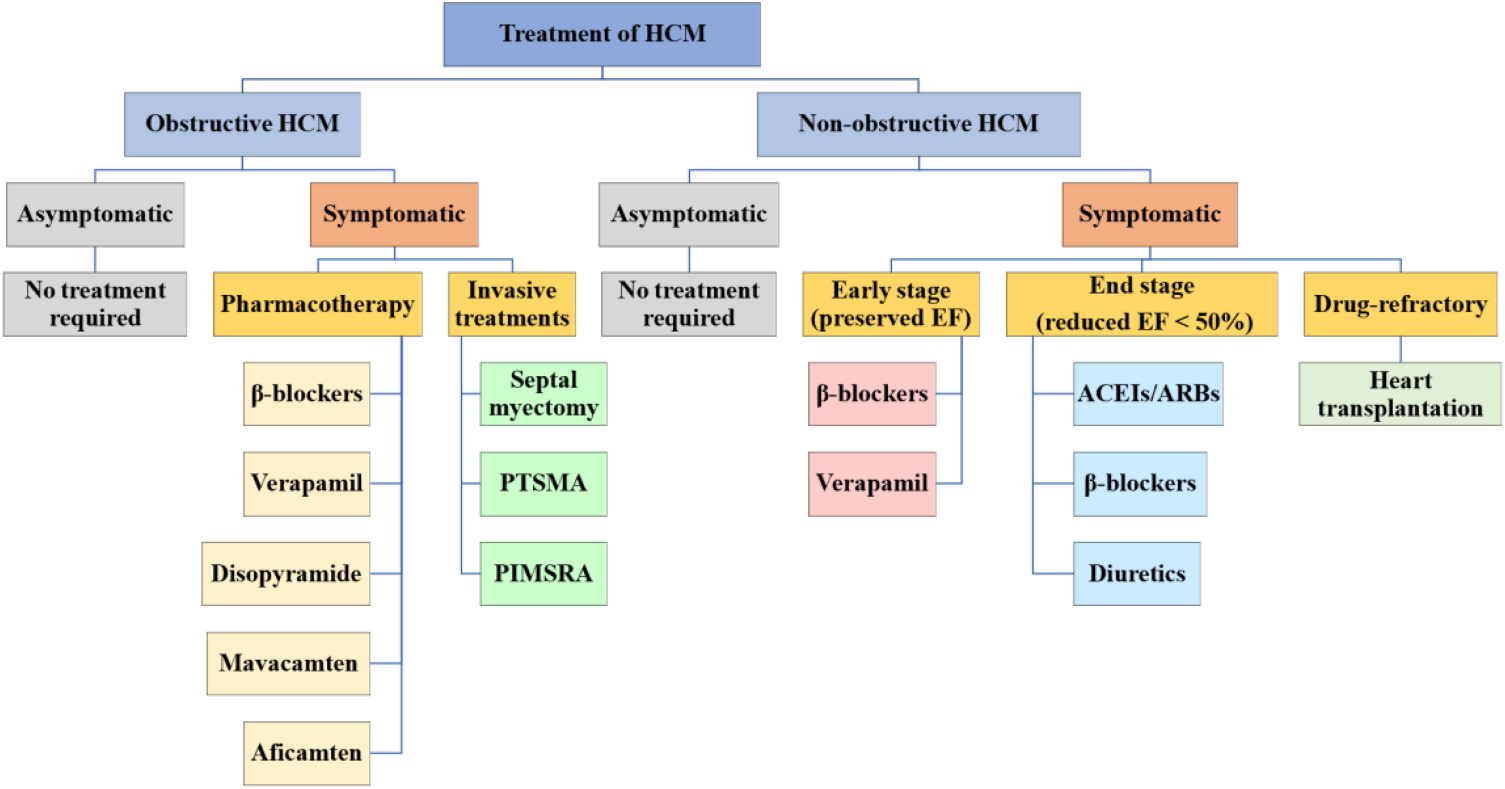
Treatment of HCM. HCM is categorized into obstructive and non-obstructive. Asymptomatic HCM generally does not require treatment and should be followed up regularly, while symptomatic HCM is treated differently for the two types. HCM, hypertrophic cardiomyopathy; PTSMA, percutaneous transluminal septal myocardial ablation; PIMSRA, percutaneous intramyocardial septal radiofrequency ablation; EF, ejection fraction; ACEIs, angiotensinconverting enzyme inhibitors; ARBs, angiotensin receptor blockers.

To date, no drug has been demonstrated to change the natural history of HCM, lessen its maximum wall thickness, or stop the disease's progression (8, 15). The main purposes of current pharmacotherapy are to alleviate clinical symptoms and enhance the quality of life (15). Therefore, asymptomatic HCM patients do not need treatment and only need regular evaluation (46, 47). Whether or not a patient has LVOTO affects the guiding principles of treatment for symptomatic patients (Figure 1) (8).

### 3.1 Patients with obstructive HCM

#### 3.1.1 Pharmacotherapy of patients with obstructive HCM

##### 3.1.1.1 Traditional drugs

**Figure 2:**
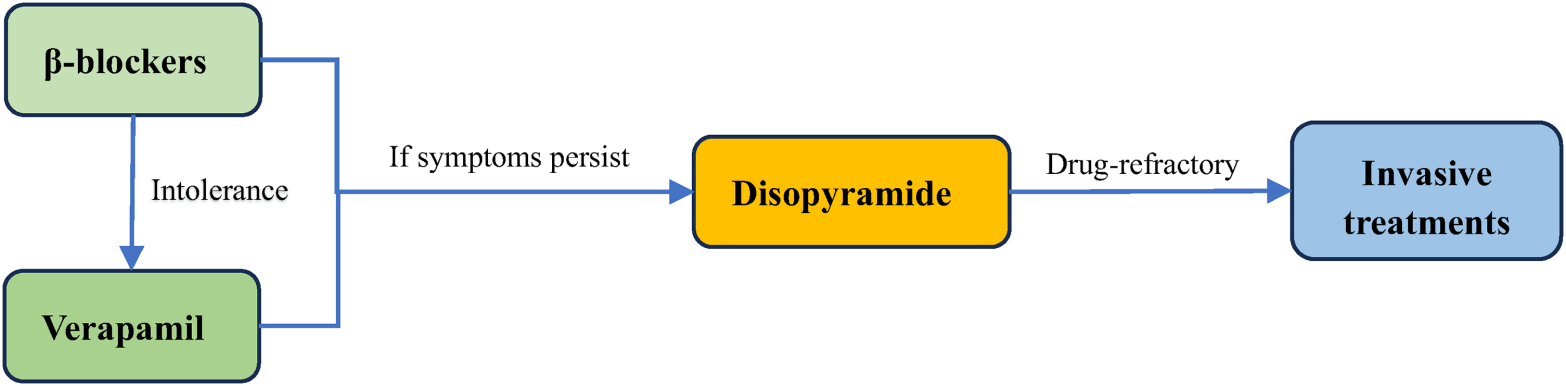
Treatment of obstructive HCM. The first line treatment for obstructive HCM is β-blockers, and they can be replaced by verapamil if the patient does not tolerate. Disopyramide can be added if symptoms persist to be unrelieved. For drug-refractory obstructive HCM, surgery is required.

In patients with symptomatic obstructive (LVOTO at resting or provoked) HCM, the β-blockers without vasodilatory effect are the first-line pharmacological treatment to improve patients’ symptoms (48, 49). The first drugs, β-blockers, which were introduced in 1962 for HCM (50), slow the heart rate, prolong ventricular filling time, and improve ventricular diastolic filling (7, 8, 35, 51). At the same time, they have the negative inotropic action that can mitigate the LVOT pressure and alleviate LVOTO (7, 52). Applied now β-blockers mainly include metoprolol, propranolol and bisoprolol, starting from a small dose and gradually titrating up to the maximum tolerated dose (48). Verapamil is suggested for symptom alleviation in cases of β-blockers intolerance (2, 3, 48). It reduces myocardial contractility and improves ventricular diastolic function by blocking Ca^2+^ channels in cardiomyocytes, which reduces LVOTO and improves patients’ symptoms (3, 53). However, Verapamil is not recommended for sinus bradycardia, sick sinus syndrome, hypotension, second- and third- degree atrioventricular block (15). If symptoms remain uncontrolled with the drugs mentioned above, disopyramide can be added to combination with verapamil or β-blockers (Figure 2) (54). Disopyramide belongs to class Ia antiarrhythmic drugs and has a strong negative inotropic effect, which can lower the contractility of the ventricle (8, 55, 56). The study by Sherrid et al. compared 118 obstructive HCM patients treated with disopyramide with 373 patients who did not receive disopyramide. At a mean follow-up of 3.1 years, 78 (66%) patients maintained on disopyramide had a decrease at resting mean LVOT pressure gradient from (75 ± 33) mmHg to (40 ± 32) mmHg, which improved symptoms and delayed the need for invasive treatments (20).

##### 3.1.1.2 Myosin inhibitors

As research into the pathophysiology of HCM continues, its treatment continues to advance. The emergence of cardiac myosin inhibitors has compensated for the shortcomings of traditional drugs that only control symptoms but do not improve cardiac function, and has brought new hope for the prognosis of patients with HCM. There are two main types of myosin inhibitors being studied, mavacamten and aficamten (57, 58).

Mavacamten which was approved recently by the FDA on April 28, 2022, for the treatment of symptomatic obstructive HCM is a small-molecule allosteric inhibitor of myosin that can target the ATPase that acts on cardiac myosin, to reversibly inhibit the actin-myosin cross-bridges (59–61). Treatment with mavacamten decreased contractility in animal models, abolished SAM, and alleviated LVOTO (62, 63). Mavacamten was tested in a non-randomized phase 2 trial (PIONEER-HCM, NCT02842242) on 21 individuals with symptomatic obstructive HCM. After cessation of background treatment, in Cohort A (*n* = 11), higher dosages (10–20 mg/d) of mavacamten led to a reduction (mean 15%) in left ventricular ejection fraction (LVEF) as early as 2 weeks into treatment, indicating that mavacamten can induce a decrease in myocardial contractility (23). This study also demonstrated that mavacamten resulted in a significant and rapid reduction in the degree of post-exercise LVOTO, as well as an increase in exercise capacity and improvement in patients’ symptoms (23). The phase 3 trial (EXPLORER-HCM, NCT03470545), a randomized, double-blind, placebo-controlled trial, also indicated the same results. Compared to the placebo group, more people (37% vs. 17%) in the mavacamten group reached the primary composite endpoint (an increase in peak oxygen consumption ≥1.5 ml/kg/min with at least a one-step decrease in NYHA classification or an increase in peak oxygen consumption ≥3.0 ml/kg/min without deterioration in NYHA classification) (59). The degree of post-exercise LVOT gradient in mavacamten treatment also had more significantly reduced (−36 mmHg, 95% CI −43.2 to −28.1; *p* < 0.0001) than those on placebo (59). In addition to this, patients in the mavacamten group showed significant improvements in exercise capacity, health condition, N-terminal pro-B-type natriuretic peptide levels and KCCQ scores compared to the placebo group (59). It likewise demonstrated that mavacamten was similarly safe and tolerable compared to placebo, with generally mild treatment-induced adverse events (59). In a clinical study (VALOR HCM, NCT04349072), it was discovered that mavacamten dramatically decreased the fraction of patients fulfilling recommended criteria for septal reduction therapy (SRT) after 16 weeks in obstructive HCM patients with unremitting symptoms (64).

Aficamten (CK-274) is the second small molecule myosin inhibitor to enter clinical trials. It has a shorter half-life than mavacamten, reaching stable concentrations within 2 weeks and a relatively wider therapeutic window (27, 58). Aficamten reduces actin-myosin cross-bridge contacts and decreases contractility by binding to myosin in a selective and reversible manner (65). It moved into human clinical trials and finished a phase 1 trial with healthy volunteers. The study has shown that aficamten is secure and well-tolerated in healthy volunteers, with a predicted human half-life suitable for once-daily dosage, reaching steady state within 2 weeks (66). In aficamten's phase 2 study, compared to placebo, aficamten led to significant LVOT gradient decreases at resting and with Valsalva, and there was a modest reduction in ejection fraction (EF) (65). In addition to this, N-terminal pro-B-type natriuretic peptide levels were significantly reduced by 62% and NYHA functional class also improved (65).

#### 3.1.2 Invasive treatments of obstructive HCM

##### 3.1.2.1 Septal myectomy

For patients with NYHA cardiac function class II-IV, or severe chest pain, or other exertional symptoms (e.g., syncope, syncope with aura) that are refractory to medications and interfere with daily activities or quality of life, and associated with LVOT gradient more than 30 mmHg at resting or 50 mmHg with physiological provocation, surgical septal myectomy is the preferred treatment option (1, 3, 67, 68). Myectomy consistently eliminates outflow obstruction immediately and permanently (while normalizing LV pressures and maintaining systolic function), and it may also reverse LA remodeling, reduce LV hypertrophy, decrease the risk of SCD and AF, as well as mitral regurgitation (1). At present, the modified Morrow procedure is commonly used, which is an extended septal excision. Resection involves the IVS, the level of the papillary muscles, and the posterior lateral free wall in addition to extending past the mitral septal contact point (15, 69). In patients with secondary mitral valve insufficiency caused by LVOTO, in the vast majority of cases, mitral valve surgery is not required, and mitral regurgitation is mostly (> 95%) eliminated after septal myectomy (10). In patients with preoperative lesions of the mitral valve itself, intervention of the mitral valve and its appendages is performed in conjunction with hypertrophic myocardial resection (15). For patients with concomitant AF, it is recommended to combine septal myectomy with the AF Maze procedure (38). The surgeon's experience and that of the entire surgical team have a significant impact on the outcome of a septal myectomy (10). In experienced high-volume HCM centers, mortality rate is less than 1%, and the success rate is 90%–95% (1, 10, 70), the long-term survival rate and age matched close of the general population (71). The same period such as mitral valve surgery, perioperative death rate is about 3%–4% (31).

##### 3.1.2.2 Percutaneous transluminal septal myocardial ablation (PTSMA)

PTSMA is a method to reduce LVOT gradient and LVOTO by injecting anhydrous alcohol into one or more septal branches of the left anterior descending branch through a catheter, causing myocardial infarction in the corresponding hypertrophic part and thinning the base of the IVS (30, 72). At present, ASA is mainly used, which is the most classic PTSMA. PTSMA has emerged as the best substitute to septal myectomy for decreasing HCM outflow gradients and improving heart failure symptoms (73, 74). Similar to septal myectomy, PTSMA should also be carried out in a high-volume center setting by a skilled HCM team (73). Septal myectomy may be preferred in young ones or when extra papillary muscle therapy or mitral valve surgery need to be performed (16). PTSMA is recommended for older patients with various coexisting illnesses and a significant surgical risk or those who are absolutely unwilling to undergo surgery (75–77). ASA, a most classic PTSMA, uses absolute alcohol (96%–99% ethanol) to chemically ablate and block the septal artery, causing regional myocardial necrosis, thus eliminating IVS hypertrophy and reducing LVOTO (11). ASA for HCM patients with indications can effectively reduce LVOT gradient and improve symptoms, increase activity tolerance, and long-term prognosis is good (8).Short hospital stays and quick recovery are advantages of ASA (29). The procedure mortality and risk of sequelae are comparable to those associated with septal myectomy (1, 8). ASA differs from septal myectomy in that it results in a less even and slower gradient decrease, a high (10%-15%) likelihood of complete heart block (CHB), which necessitates permanent pacing, repeats treatments, and a potential elevated risk of scar-related ventricular arrhythmias (15, 16, 78). Furthermore, the septal coronary architecture restricts the success of ASA (79–81).

##### 3.1.2.3 Percutaneous intramyocardial septal radiofrequency ablation (PIMSRA)

Unfortunately, septal coronary architecture limits the effectiveness of ASA despite the fact that it can undoubtedly relieve clinical symptoms and lower the LVOT gradient (34, 79). To take advantage of ASA and protect the subendocardial conduction system, a novel procedure (named the Liwen procedure) was proposed, whereby PIMSRA is used in the hypertrophic IVS to reduce LVOTO (32). PIMSRA is an ultrasound-guided, non-stopping cardiac procedure in which a radiofrequency (RF) needle is delivered directly to the IVS hypertrophy site through the skin, intercostal space, and apical region of the heart with precise puncture. The high-frequency alternating current emitted from the front end of the RF electrode needle causes localized heating of the hypertrophied myocardial tissue and dehydration of the myocardial cells, resulting in irreversible coagulative necrosis. At the same time, it can make the septal branch in the ablated myocardium coagulate to form a reaction band, thus blocking the blood supply of the hypertrophied myocardial tissue, and eventually thinning the thickness of the IVS and widening the inner diameter of the stenosis, thus relieving the obstruction (32, 34, 79, 82). Medium-term follow-up has shown that PIMSRA can effectively reduce the IVS thickness, lower LVOT gradient or heart cavity pressure difference and improve the patients’ symptoms and quality of life, but relevant experiences and long-term follow-up data are limited (34, 79, 82).

### 3.2 Patients with non-obstructive HCM

The majority of patients with non-obstructive HCM are asymptomatic or very minimally symptomatic (NYHA cardiac function class I–II), and they typically have a low risk of developing progressive heart failure or other negative effects, no pharmacological therapy required. They need to start clinical observation and follow-up, and the assessment for risk of SCD is also necessary (2). The common symptoms of non-obstructive HCM include exertional dyspnea and chest pain, mainly due to diastolic dysfunction in the early stage (preserved EF) (15). Verapamil and β-blockers can still be used to improve symptoms (83). When entering the stage of advanced heart failure (reduced EF <50%), NYHA functional classes III/IV, if systolic dysfunction is present, angiotensin-converting enzyme inhibitors (ACEIs), angiotensin II receptor blockers (ARBs), β-blockers, and diuretic agents can be used (15). Only a small percentage of patients will progress to the rare, very symptomatic, advanced stage of the disease with persistent, drug-refractory low output heart failure symptoms that require consideration of heart transplantation (Figure 1) (84).

Maintaining sinus rhythm and controlling ventricular rate in non-obstructive HCM patients with AF are keys to treatment, and such patients should undergo anticoagulation without the need for a CHA_2_DS_2_-VASc score because of the high risk of stroke (48, 54, 84, 85). Lifetime anticoagulation is generally required, even if the sinus rhythm has recovered (2).

### 3.3 Gene therapies of HCM

Traditionally, HCM is considered to be the most common single-gene disease, but in recent years, some studies have found that a small number of patients have compound or polygenic mutations, and about 60% of HCM patients can be found to have a clear causative mutation by genetic testing (2, 86). With technological and cognitive advances, gene therapy may become a viable treatment option for HCM patients. Since most of the mutations occur in the MYH7 and MYBPC3 genes, most of the new gene therapies have focused on these two genes (86–90).

#### 3.3.1 Exon skipping

Exon skipping is the removal of exons containing defective genes by selective gene splicing, resulting in reduced or no expression of the defective gene, thereby producing shorter but functional protein (87). Gedicke-Hornung et al. successfully and transiently eliminated cardiac dysfunction and prevented LVH in newborn mice with mutations in the MYBPC3 gene by exon skipping (91). It suggests that exon skipping technology is promising when applying to the treatment of HCM.

#### 3.3.2 RNA trans-splicing

RNA trans-splicing refers to splicing two independently transcribed RNAs together, so that they can be repaired and transcribed into normal proteins (92). However, in the available studies, despite relative success *in vitro*, only very few RNAs were repaired *in vivo* experiments in mice, and not enough to prevent cardiac hypertrophy (93). Therefore, further improvement of the efficiency of trans-splicing is the focus of the next research.

#### 3.3.3 Gene replacement

Gene replacement refers to the introduction of a missing gene into a specific cell so that it can be re-expressed. It is usually used in cases where a gene mutation results in reduced or absent protein levels, such as the MYBPC3 gene mutation (94, 95). Mearini et al. injected the MYBPC3 gene into MYBPC3 knockout mice via the adeno-associated virus 9 (AAV9) vector and found that MYBPC3 expression suppressed the accumulation of mutant mRNAs (96). Subsequently, Prondzynski et al. demonstrated the successful expression and suppression of cardiac hypertrophy when the gene was introduced into MYBPC3-mutant cardiomyocytes and human embryonic stem cell lines (93).

#### 3.3.4 Allele-specific silencing

Allele-specific silencing is the organism-specific silencing of genes to reduce their expression, caused by the induction of transcription of double-stranded RNA homologous to the target gene (95). Jianming Jiang et al. successfully demonstrated that in Myh6 mutation mice, an RNAi cassette delivered by the AAV silenced mutant allele and delayed the progression of myocardial hypertrophy (97).This technology has been shown to be an option for the treatment of autosomal dominant disorders (98).

#### 3.3.5 Gene editing

Gene editing is the production of DNA double-strand breaks on specific sequences by genetically engineered modified nucleases, inducing the organism to repair them by non-homologous end joining (NHEJ) or homologous recombination (HR) (95). Jiali Nie et al. demonstrated that *in vivo* genome editing of MYBPC3 using the CRISPR/Cas9 system in 1098hom rats partially restored MYBPC3 protein expression and attenuated cardiac function (99). This suggests that CRISPR/Cas9-based gene editing technology has great potential for the treatment of HCM.

## 4 Dilemma and future development of HCM

This article elaborates recent development in pharmacotherapy, invasive treatments, gene therapies, and the current predicament, which provides certain guidance and inspiration for the future research direction and development. Although significant progress has been made in the treatment of HCM, and currently available treatment methods for HCM have substantially reduced patient mortality, the treatments of obstructive and non-obstructive HCM still have significant unmet needs. There is still no complete cure for HCM. The existing drug therapy can improve patients’ clinical symptoms and exercise ability, but there is almost no proof to prove it can alter the natural history of HCM patients (8). For drug-refractory obstructive HCM, septal myectomy and ASA are presently regarded as standard therapeutic techniques. However, septal myectomy requires extracorporeal circulation and ASA may harm the conduction pathway found beneath the endocardium (15, 16). AF is the most frequent arrhythmia complicating HCM. Maintaining sinus rhythm in HCM patients can help improve diastolic function, reduce the risk of stroke, and improve overall survival. However, the pathogenesis, prevention, clinical use of drugs, and surgical methods of AF in patients with HCM need to be further investigated. New medications and minimally invasive treatments have begun to appear in the past few years, and they have the possibility to drastically change the therapeutic panorama. There is a considerable prospect for the myosin inhibitors and minimally-invasive procedures. Data from these therapies’ initial trials indicate promising outcomes, but longer-term safety and effectiveness data are required. In one study, soluble epoxide hydrolase antagonists improved cardiac diastolic function and may also have future applications in the treatment of HCM (100). Surgical interventions keep going to develop, while innovative methods like RF myocardial ablation, transcatheter mitral valve repair, and high intensity focused ultrasound may eventually provide substitutes for patients with obstructive HCM. For non-obstructive HCM, the unmet therapeutic need is primarily for pharmacologic therapy in symptomatic patients, especially those with restrictive physiology and preserved EF, to slow progression to end stage HF. This is the main therapeutic issue to be addressed in the future. Gene therapy is expected to be a cure for HCM from the root cause, but it is still in the basic stage and there are many problems to be solved. For example, currently, AAV is mostly used as a vector for gene therapy, because it is less pathogenic. However, in a large proportion of patients, it may stimulate the body to produce neutralizing antibodies, which may lead to treatment failure (101). Besides, the ethical issues cannot be ignored before gene therapy enters clinical trials. In addition, some HCM patients are still unable to clearly detect the causative genes. Understanding the causative genes and pathogenesis of HCM so as to cure HCM from the root of pathophysiological mechanism will become the future research direction in this field. It is believed that gene-guided precision medicine will have the potential to completely treat HCM in the future.
